# Effect of thimerosal, methylmercury, and mercuric chloride in Jurkat T Cell Line

**DOI:** 10.2478/v10102-012-0026-1

**Published:** 2012-09

**Authors:** Gianpaolo Guzzi, Paolo D. Pigatto, Francesco Spadari, Caterina A.M. La Porta

**Affiliations:** 1 Italian Association for Metals and Biocompatibility Research – A.I.R.M.E.B., Via A. Banfi, 4, 20122 Milan, Italy; 2 Department of Technology for Health, Dermatological Clinic, IRCCS Galeazzi Hospital, University of Milan, Milan, Italy; 3 Department of Biomedical, Surgical and Dental Sciences, Unit of Oral Pathology and Medicine, Ospedale Maggiore Policlinico Fondazione Ca’ Granda IRCCS, University ofMilan, Milan, Italy; 4 Department of Biosciences, University of Milan, Milan, Italy

**Keywords:** Cell Survival/drug effects, organic mercury compounds, mitochondrial membranes/drug effects, MTT, T-Lymphocytes/drug effects, Cell Death/drug effects

## Abstract

Mercury is a ubiquitous environmental toxicant that causes a wide range of adverse health effects in humans. Three forms of mercury exist: elemental, inorganic and organic. Each of them has its own profile of toxicity. The aim of the present study was to determine the effect of thimerosal, a topical antiseptic and preservative in vaccines routinely given to children, methyl mercury, and mercuric chloride on cellular viability measured by MTT in Jurkat T cells, a human T leukemia cell line. The treatment of Jurkat T cells with thimerosal caused a significant decrease in cellular viability at 1 μM (25%, *p<*0.05; IC50: 10 μM). Methyl mercury exhibited a significant decrease in cellular viability at 50 μM (33%, *p<*0.01; IC50: 65 μM). Mercuric chloride (HgCl_2_) did not show any significant change in cellular survival. Our findings showed that contrary to thimerosal and methyl mercury, mercuric chloride did not modify Jurkat T cell viability.

## Introduction

Mercury, one of the most widely diffused and hazardous organ-specific environmental contaminants, exists in a wide variety of physical and chemical states, each with unique characteristics of target organ specificity (Aleo *et al.*, 2002). Mercury occurs in three forms: the elemental or metallic form, inorganic salts, and organic compounds. The toxicity of mercury is complex and depends on the form of mercury, route of entry, dosage, and age at exposure (Clarkson, 1997). The organic form of mercury, mainly methyl mercury, is known to be more toxic than the inorganic form (Shenker *et al.*, 1992). Chronic exposure to low levels of methyl mercury can modulate T- and B-cell functions (cytokine production, cell growth, and proliferation) and different cellular processes leading to apoptotic cell death (Makani *et al.*, 2002; Shenker *et al.*, 1992). Ethyl mercury is an organic mercury compound, and in the form of thimerosal has been used as a topical antiseptic and as a preservative in vaccines routinely given to children, including diphtheria-tetanus-acellular pertussis (DTP), hepatitis B, and some Haemophilus influenzae type B (Goldman & Shannon, 2001; Halsey, 1999; Pichichero *et al.*, 2002). Thimerosal (as sodium ethylmercuric thiosalicylate) contains 49.6% mercury by weight and is metabolized to ethyl mercury and thiosalicylate. The normal dose of a pediatric vaccine contains about 12.5–25 μg of mercury per 0.5 ml. (No authors listed, AAP, 1999). Massive overdoses from inappropriate use of products containing thimerosal have resulted in toxic effects (Axton, 1972; Fagan *et al.*, 1977; Lowell *et al.*, 1996; Matheson *et al.*, 1980; Pelassy *et al.*, 1994; Pfab *et al.*, 1996). Inorganic mercury (I-Hg) compounds (as mercury salts) are also a significant source of mercury overexposure in both adults and children in some countries (Clarkson, 2002). Inorganic mercury compounds have been used for many years in numerous products, including various medications, germicidal soaps, teething powders, and skin lightening cream containing mercury (Clarkson, 2002). Many of these mercury-based products are still in use today (Geier *et al.*, 2010; Goldman & Shannon, 2001). In the present study, we evaluated the effect of thimerosal, methyl mercury and mercuric chloride (HgCl_2_) on the viability of Jurkat T cells by (3-(4,5-Dimethylthiazol-2-yl)-2,5-diphenyltetrazolium bromide (MTT) assay.

## Methods

### Cell culture

Human T leukemic Jurkat cells were purchased from American Type Culture Center (ATCC no. TIB-152) (Rockville, MD, USA) and maintained in RPMI-1640 medium supplemented with 10% fetal bovine serum, 1% glutamine, and 1% antibiotics/antimicotics (pen./strep.). The cells were grown at 37°C in a humidified atmosphere of 5% CO_2_.

### Mercury and its chemical compounds

Thimerosal (EtHg), methyl mercury (MeHg) and mercuric chloride [(mercuric (II) chloride (HgCl_2_) also termed ‘mercury two’)] were purchased from Sigma. PBS and water were used to dilute mercuric chloride (HgCl_2_) and thimerosal, respectively. Cells treated only with vehicles were used as controls.

### Cytotoxicity assay (MTT)

The principle behind this technique depends on the capacity of living cells to reduce tetrazolium salt [3-(4,5-dimethylthiazol-2-yl)-2,5-diphenyltetrazolium bromide] to a formazan crystal in their metabolizing mitochondria. The number of 1×10^4^ cells/well Jurkat T cells (ATCC no. TIB-152) were seeded into 96 well plates and exposed to thimerosal, methyl mercury, and mercuric chloride (HgCl_2_) at concentrations of thimerosal (0.01-0.1-1-10-50-100-250 μM), methyl mercury (30-50-80-100-250 μM), and mercuric chloride (HgCl_2_ (20-40-60-80-100 μM). The plates were incubated at 37°C in a humidified atmosphere containing 5% CO_2_. After 48 hours, the medium was discarded and 20 μl/well of MTT solution (5 mg/ml) was added and incubated for 3 hours at 37°C (5% CO_2_). Finally, 20μl/well of isopropanol was added and the color intensity was read spectrophotometrically at 590 nm using a Microplate Reader (Bio-Rad Model 550, California, USA).

### Statistical analysis

The ANOVA one-way test was used to determine statistical significance. A *p-*value of less than 0.05 was considered to be statistically significant.

## Results

**Figure 1:**
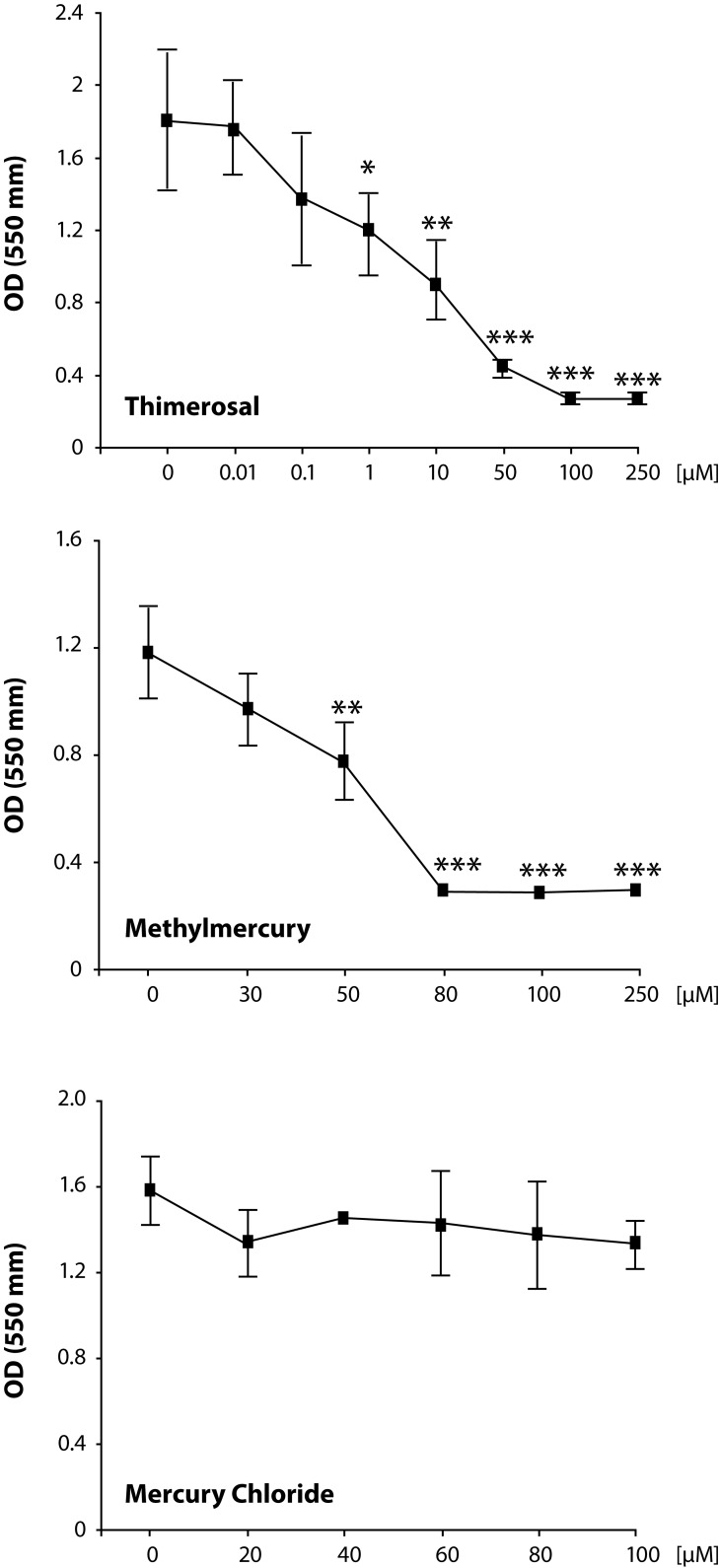
Viability of Jurkat cells determined by MTT-assay after exposure to thimerosal, methylmercury and mercury chloride. The data are expressed as mean ± SEM. Asterisks indicate significant differences from control cells without treatment (n=3, *p<0.05; **p<0.01; ***p<0.001). Note, the x-axis is not a linear scale.

We exposed Jurkat T cells to thimerosal, methyl mercury and mercuric chloride in the concentrations reported in Figure 1 for 48 hours. Upon exposure to thimerosal, methyl mercury and mercuric chloride (HgCl_2_), the viability of cells was measured with MTT assay. As shown in Figure 1, the treatment of Jurkat T cells with thimerosal caused a significant decrease in cellular viability at 1μM (25%, *p<*0.05; IC50: 10μM). Methyl mercury exhibited a significant decrease in cellular viability at 50μM (33%, *p<*0.01; IC50: 65μM). Finally, at all concentrations analyzed, mercuric chloride (HgCl_2_), did not show any significant change in cellular survival (Figure 1).

## Discussion

Mercury is ubiquitous in the environment and exposure occurs from the use of mercury-containing dental amalgam, vaccine preservatives, and ingestion of fish containing high levels of methyl mercury (Counter & Buchanan, 2004; Krantz & Dorevitch, 2004; Ratcliffe *et al.*, 1996). In the literature, however, there are few data showing the effect of organic and inorganic mercury on cell viability. Considerable concern has been expressed recently over the cumulative dose of ethyl mercury given to children through routine immunizations (Geier *et al.*, 2010; Hornig *et al.*, 2004). The source of mercury in vaccines is the antimicrobial preservative thimerosal, containing 49.9% mercury by weight. Our findings demonstrate that thimerosal at the concentration usually found in vaccines, affects significantly cellular viability. A recent paper showed that after thimerosal exposure at the same concentration as tested in the present study, a human glioblastoma cell line displayed a similar effect (James *et al.*, 2005). On the other hand, the form of mercury that accumulates in the food chain is methyl mercury. Some people may be exposed to higher levels of mercury in the form of methyl mercury if they have a diet high in fish, shellfish, or marine mammals that come from mercury-contaminated waters. Colombo *et al.* (2004) determined the sensitivity of Jurkat T cells to up to 1 μM of methyl mercury after 48 hours of exposure (Colombo *et al.*, 2004). They found that cellular viability determined by MTT assay showed no toxic effects during the first 48 hours, yet exposure for up to 72 hours caused a significant decrease in cellular viability at the higher dose of mercury (1 μM) (Pelassy *et al.*, 1994). Our findings are in accordance with these data and show that organic mercury, such as methyl mercury and thimerosal, are more cytotoxic than inorganic mercury (as HgCl_2_). Experiments are in progress to ascertain the underlying mechanisms of ethyl mercury induced cell death. It has been proposed to induce depletion of thiol reserves (*e.g.*: GSH) and ROS damage, activating death-signaling pathways (Makani *et al.*, 2002). A previous study showed that thimerosal was able to induce apoptosis and G2/M phase in human leukemia U937 cells (Woo *et al.*, 2006). Finally, according to other authors (Bahia *et al.*, 1999; Ogura *et al.*, 1996), methyl mercury showed a higher toxicity compared to mercuric chloride (HgCl_2_). Recently, mercuric chloride (HgCl_2_) was reported to affect the differentiative capacity instead of proliferation in neural stem cells (Cedrola *et al.*, 2003). Further studies will attempt to assess the possible effect of thimerosal as preservative in vaccines. Our data showed an effect of organic mercury on the viability of Jurkat T cells, suggesting a possible toxic effect of these compounds of mercury *in vivo*.
